# Primary pulmonary adenocarcinoma in a 16-year-old boy – a five-year follow-up

**DOI:** 10.3402/ecrj.v3.32633

**Published:** 2016-11-07

**Authors:** Ane Stillits Måreng, Seppo W. Langer, Uffe Bodtger

**Affiliations:** 1 Department of Respiratory Medicine, Naestved Hospital, Naestved, Denmark; 2 Department of Oncology, Head, Thoracic and Neuroendocrine Unit, Copenhagen University Hospital, Rigshospitalet, Copenhagen, Denmark; 3 Institute of Regional Health Research, University of Southern Denmark, Odense, Denmark

**Keywords:** non-small cell lung cancer, metastasis, survival, adolescent, diagnosis, survival

## Abstract

Primary pulmonary adenocarcinoma in children or adolescents is a rare disease, and as such, there are no randomised studies on lung cancer for this age group. Treatment choice is extrapolated from studies in adults (mean age of participants: 60 years). We present the 5-year follow-up of a 16-year-old boy who presented with metastatic primary pulmonary adenocarcinoma (T3N3M1a) and was treated aggressively, including radiation therapy for local and distant recurrence. He had complete remission, had completed his education, was employed full-time, and suffered only from mild side effects to treatment.

A 16-year-old Caucasian boy was admitted to our unit in January 2011 with a 2-month history of shortness of breath during exercise, haemoptysis, loss of appetite, and a weight loss of 3 kg. Labour, birth, and childhood had been unremarkable. There was no history of no tobacco smoke exposure, previous childhood cancer, environmental exposure to known cancerogenics, or first- or second-generation relatives with cancer. He attended ninth grade, was active with sports, and lived in a rural town with his middle-class family. A chest x-ray showed a mass in right lower lobe (RLL). At admission, he presented with normal respiratory frequency, was slim but not underweight, and a fixed bronchial obstruction at auscultation in IC4 of the anterior left hemithorax.

A chest CT demonstrated two RLL lesions (T3), ipsi- and contralateral mediastinal lymphadenopathy (N3), and an intraluminal lesion obstructing left main bronchus (M1a) (Fig. 1). Bronchoscopy and endobronchial ultrasound confirmed Tumor-Node-Metastasis (TNM) stage: primary pulmonary adenocarcinoma with no signs of a foetal subtype (positive immunohistochemical staining to cytokeratine-7, TTF-1, and napsin with solitary cells additionally positive to cytokeratin-5, P63, and vimentin; all negative to placental alkaline phosphatase and synaptophysin). The patient underwent immediate bronchoscopic laser resection of tumour in the left main bronchus with immediate relief of dysponea. Extensive histopathologically workup showed no mutations of EGFR, KRAS, or BRAF, no expression of ALK protein, no ALK-EML4 rearrangement, and no ROS1 translocation. Stage was confirmed by positron emission tomography (PET)-CT.

**Fig. 1:**
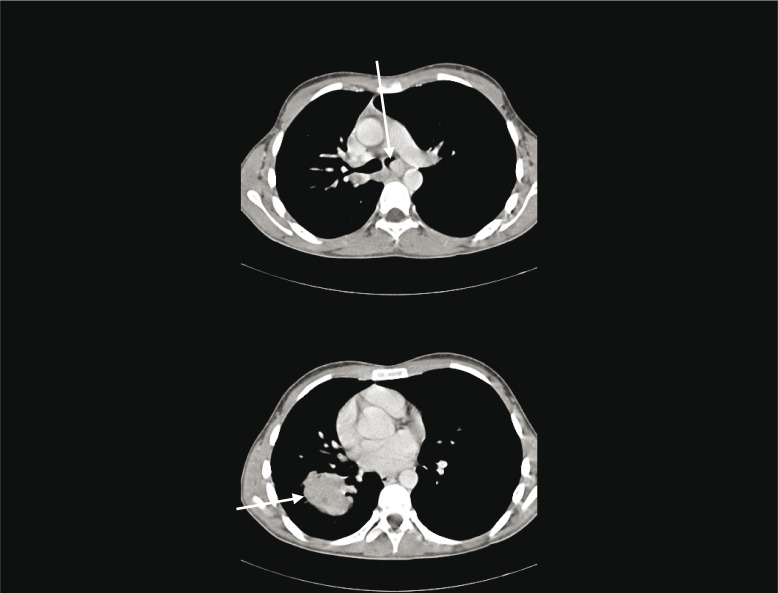
Contrast-enhanced chest CT at diagnosis. Arrows show tumour mass in left main bronchus (upper image) and in right lower lobe (lower image).

Downstaging was obtained with chemotherapy (nine courses of cisplatin, vinorelbine plus bevacizumab), thoracic radiation therapy (66 Gy on 33 fractions, 5 fractions/week), and additional three courses of platinum doublet.

An FDG PET-CT scan showed complete regression of the left tumour. In April 2012, RLL was resected. Tumour islets identical with primary cancer were found in stations 7 and 12R. Postoperatively, he received adjuvant platinum-based chemotherapy.

Next-generation sequencing (Formalin-Fixed, Paraffin-Embedded tissue (FFPE)-purified genomic DNA; Ion AmpliSeq Cancer Hotspot Panel version 2) of the resected tumour revealed a non-targetable mutation in KIT gene (c.1676T >A, p.V559D). The other 49 genes were without mutations: ABL1, EZH2, JAK3, PTEN, AKT1, FBXW7, IDH2, PTPN11, ALK, FGFR1, KDR, RB1, APC, FGFR2, RET, ATM, FGFR3, KRAS, SMAD4, BRAF, FLT3, MET, SMARCB1, CDH1, GNA11, MLH1, SMO, CDKN2A, GNAS, MPL, SRC, CSF1R, GNAQ, NOTCH1, STK11, CTNNB1, HNF1A, NPM1, TP53, EGFR, HRAS, NRAS, VHL, ERBB2, IDH1, PDGFRA, ERBB4, JAK2, and PIK3CA.

An FDG PET-CT scan was performed every 3 months. In October 2014, an FDG-positive mediastinal lesion was assessed by mediastinoscopy: no malignancy.

In April 2015, he underwent surgery for a solitary, symptomatic cerebral metastasis, followed by stereotactic radiation therapy (18 Gy×1). In June 15, a biopsy-confirmed mediastinal and cervical lymph node recurrence was treated with nine series of docetaxel: partial remission after three courses and complete remission after nine courses. He continued clinical and PET-CT assessments every 3 months.

At 5-year follow-up (January 2016), he was without evidence of new recurrence. He had finished his commercial education and started an employment at a worldwide pharma industry. He had mild peripheral lower limb neuropathy as only side effect to treatment.

## Discussion

The incidence of primary lung cancer in persons under 18 years is less than 0.05/100,000 persons, and primary pulmonary adenocarcinoma constitutes <10% of all cases (1–4). Benign or secondary malignant lesions in the lung are much more prevalent, though still rare. No specific oncogenic drivers, mutations, genes, tumour antigens, or other characteristics have been found in children or adolescents with primary pulmonary adenocarcinoma, though most patients have metastatic disease at presentation, and the 5-year survival is 25% (1, 2, 4). No randomised clinical trials on childhood primary lung cancer have been performed: almost an orphan disease, treatment strategies are extrapolated from studies conducted in older populations. In published reports on phase III-studies on first- or second-line therapy of unresectable lung adenocarcinoma, median age was 60 years, and less than 1% was below 30 years (5–19). Future studies on paediatric lung cancer will probably focus on genetic promoter mutations, as survival is improved when EGFR or KRAS mutations are present (20). Our patient had no targetable mutations.

An increasingly aggressive approach to advanced or metastatic non-small cell lung cancer (NSCLC) has emerged during the last years: intended curative chemoradiotherapy in locally advanced stages (21, 22), and intended curative surgery in M1b oligometastatic NSCLC with single brain metastasis and a small primary lesion (23). Our patient was offered extensive chemotherapy modified from guidelines for adult patients with stage IV primary pulmonary adenocarcinoma (24), and was successfully downstaged as assessed by both Response Evaluation Criteria In Solid Tumors (RECIST) criteria and invasive tissue sampling. Using an aggressive approach including surgical resection of single metastases, our patient had no signs of new relapse at the control visit 5 years after diagnosis and had only a minimum of persisting side effects.
